# Supplementary material for: HIVprotI: an integrated web based platform for prediction and design of HIV proteins inhibitors
Source: J Cheminform. 2018 Mar 9;10:12. doi: 10.1186/s13321-018-0266-y (PMC5845081; doi:10.1186/s13321-018-0266-y)
Supplement: Supplementary file 2 — Additional file 2. Source code of HIVProtI web server. [file 13321_2018_266_MOESM2_ESM.zip › HIVprotI_Source-code/hivprotI/weka-3-6-6/doc/weka/gui/beans/package-summary.html]

weka.gui.beans


---


|  |  |  |
| --- | --- | --- |
| **Overview** \| **Package** \| Class \| **Tree** \| **Deprecated** \| **Index** \| **Help** \| **Weka's home** | |  |
| **PREV PACKAGE**   **NEXT PACKAGE** | **FRAMES**    **NO FRAMES**     **All Classes** |  |


---

## Package weka.gui.beans

| **Interface Summary** | |
| --- | --- |
| **BatchClassifierListener** | Interface to something that can process a BatchClassifierEvent |
| **BatchClustererListener** | Interface to something that can process a BatchClustererEvent |
| **BeanCommon** | Interface specifying routines that all weka beans should implement in order to allow the bean environment to exercise some control over the bean and also to allow the bean to exercise some control over connections. |
| **ChartListener** | Interface to something that can process a ChartEvent |
| **ConnectionNotificationConsumer** | Interface for Beans that can receive (dis-)connection events generated when (dis-)connecting data processing nodes in the Weka KnowledgeFlow. |
| **CustomizerCloseRequester** | Customizers who want to be able to close the customizer window themselves can implement this window. |
| **CustomizerClosingListener** |  |
| **DataFormatListener** | Listener interface that customizer classes that are interested in data format changes can implement. |
| **DataSink** | Indicator interface to something that can store instances to some destination |
| **DataSource** | Interface to something that is capable of being a source for data - either batch or incremental data |
| **DataSourceListener** | Interface to something that can accept DataSetEvents |
| **EventConstraints** | Interface for objects that want to be able to specify at any given time whether their current configuration allows a particular event to be generated. |
| **GraphListener** | Describe interface `TextListener` here. |
| **IncrementalClassifierListener** | Interface to something that can process a IncrementalClassifierEvent |
| **InstanceListener** | Interface to something that can accept instance events |
| **LogWriter** | Interface to be implemented by classes that should be able to write their own output to the Weka logger. |
| **Startable** | Interface to something that is a start point for a flow and can be launched programatically. |
| **StartUpListener** | Interface to something that can be notified of a successful startup |
| **StructureProducer** | Interface for something that can describe the structure of what is encapsulated in a named event type as an empty set of Instances (i.e. |
| **TestSetListener** | Interface to something that can accpet test set events |
| **TestSetProducer** | Interface to something that can produce test sets |
| **TextListener** | Interface to something that can process a TextEvent |
| **ThresholdDataListener** | Interface to something that can accept ThresholdDataEvents |
| **TrainingSetListener** | Interface to something that can accept and process training set events |
| **TrainingSetProducer** | Interface to something that can produce a training set |
| **UserRequestAcceptor** | Interface to something that can accept requests from a user to perform some action |
| **Visible** | Interface to something that has a visible (via BeanVisual) reprentation |
| **VisualizableErrorListener** | Interface to something that can accept VisualizableErrorEvents |
| **WekaWrapper** | Interface to something that can wrap around a class of Weka algorithms (classifiers, filters etc). |

| **Class Summary** | |
| --- | --- |
| **AbstractDataSink** | Abstract class for objects that store instances to some destination. |
| **AbstractDataSinkBeanInfo** | Bean info class for the AbstractDataSink |
| **AbstractDataSource** | Abstract class for objects that can provide instances from some source |
| **AbstractDataSourceBeanInfo** | Bean info class for AbstractDataSource. |
| **AbstractEvaluator** | Abstract class for objects that can provide some kind of evaluation for classifier, clusterers etc. |
| **AbstractTestSetProducer** | Abstract class for TestSetProducers that contains default implementations of add/remove listener methods and defualt visual representation. |
| **AbstractTestSetProducerBeanInfo** | BeanInfo class for AbstractTestSetProducer |
| **AbstractTrainAndTestSetProducer** | Abstract base class for TrainAndTestSetProducers that contains default implementations of add/remove listener methods and defualt visual representation. |
| **AbstractTrainAndTestSetProducerBeanInfo** | Bean info class for AbstractTrainAndTestSetProducers |
| **AbstractTrainingSetProducer** | Abstract class for TrainingSetProducers that contains default implementations of add/remove listener methods and default visual representation |
| **AbstractTrainingSetProducerBeanInfo** | BeanInfo class for AbstractTrainingSetProducer |
| **Associator** | Bean that wraps around weka.associations |
| **AssociatorBeanInfo** | BeanInfo class for the Associator wrapper bean |
| **AssociatorCustomizer** | GUI customizer for the associator wrapper bean |
| **AttributeSummarizer** | Bean that encapsulates displays bar graph summaries for attributes in a data set. |
| **AttributeSummarizerBeanInfo** | Bean info class for the attribute summarizer bean |
| **BatchClassifierEvent** | Class encapsulating a built classifier and a batch of instances to test on. |
| **BatchClustererEvent** | Class encapsulating a built clusterer and a batch of instances to test on. |
| **BeanConnection** | Class for encapsulating a connection between two beans. |
| **BeanInstance** | Class that manages a set of beans. |
| **BeanVisual** | BeanVisual encapsulates icons and label for a given bean. |
| **ChartEvent** | Event encapsulating info for plotting a data point on the StripChart |
| **ClassAssigner** | Bean that assigns a class attribute to a data set. |
| **ClassAssignerBeanInfo** | BeanInfo class for the class assigner bean |
| **ClassAssignerCustomizer** | GUI customizer for the class assigner bean |
| **Classifier** | Bean that wraps around weka.classifiers |
| **ClassifierBeanInfo** | BeanInfo class for the Classifier wrapper bean |
| **ClassifierCustomizer** | GUI customizer for the classifier wrapper bean |
| **ClassifierPerformanceEvaluator** | A bean that evaluates the performance of batch trained classifiers |
| **ClassifierPerformanceEvaluatorBeanInfo** | Bean info class for the classifier performance evaluator |
| **ClassValuePicker** |  |
| **ClassValuePickerBeanInfo** | BeanInfo class for the class value picker bean |
| **ClassValuePickerCustomizer** |  |
| **Clusterer** | Bean that wraps around weka.clusterers |
| **ClustererBeanInfo** | BeanInfo class for the Clusterer wrapper bean |
| **ClustererCustomizer** | GUI customizer for the Clusterer wrapper bean |
| **ClustererPerformanceEvaluator** | A bean that evaluates the performance of batch trained clusterers |
| **ClustererPerformanceEvaluatorBeanInfo** | Bean info class for the clusterer performance evaluator |
| **CostBenefitAnalysis** | Bean that aids in analyzing cost/benefit tradeoffs. |
| **CostBenefitAnalysisBeanInfo** | Bean info class for the cost/benefit analysis |
| **CrossValidationFoldMaker** | Bean for splitting instances into training ant test sets according to a cross validation |
| **CrossValidationFoldMakerBeanInfo** | BeanInfo class for the cross validation fold maker bean |
| **CrossValidationFoldMakerCustomizer** | GUI Customizer for the cross validation fold maker bean |
| **DataSetEvent** | Event encapsulating a data set |
| **DataVisualizer** | Bean that encapsulates weka.gui.visualize.VisualizePanel |
| **DataVisualizerBeanInfo** | Bean info class for the data visualizer |
| **Filter** | A wrapper bean for Weka filters |
| **FilterBeanInfo** | Bean info class for the Filter bean |
| **FilterCustomizer** | GUI customizer for the filter bean |
| **FlowRunner** | Small utility class for executing KnowledgeFlow flows outside of the KnowledgeFlow application |
| **FlowRunner.SimpleLogger** |  |
| **GraphEvent** | Event for graphs |
| **GraphViewer** | A bean encapsulating weka.gui.treevisualize.TreeVisualizer |
| **GraphViewerBeanInfo** | Bean info class for the graph viewer |
| **IncrementalClassifierEvaluator** | Bean that evaluates incremental classifiers |
| **IncrementalClassifierEvaluatorBeanInfo** | Bean info class for the incremental classifier evaluator bean |
| **IncrementalClassifierEvaluatorCustomizer** | GUI Customizer for the incremental classifier evaluator bean |
| **IncrementalClassifierEvent** | Class encapsulating an incrementally built classifier and current instance |
| **InstanceEvent** | Event that encapsulates a single instance or header information only |
| **InstanceStreamToBatchMaker** | Bean that converts an instance stream into a (batch) data set. |
| **InstanceStreamToBatchMakerBeanInfo** | BeanInfo class for the InstanceStreamToBatchMaker bean |
| **KnowledgeFlow** | Startup class for the KnowledgeFlow. |
| **KnowledgeFlowApp** | Main GUI class for the KnowledgeFlow. |
| **Loader** | Loads data sets using weka.core.converter classes |
| **LoaderBeanInfo** | Bean info class for the loader bean |
| **LoaderCustomizer** | GUI Customizer for the loader bean |
| **LogPanel** | Class for displaying a status area (made up of a variable number of lines) and a log area. |
| **Messages** | Messages. |
| **MetaBean** | A meta bean that encapsulates several other regular beans, useful for grouping large KnowledgeFlows. |
| **ModelPerformanceChart** | Bean that can be used for displaying threshold curves (e.g. |
| **ModelPerformanceChartBeanInfo** | Bean info class for the model performance chart |
| **PredictionAppender** | Bean that can can accept batch or incremental classifier events and produce dataset or instance events which contain instances with predictions appended. |
| **PredictionAppenderBeanInfo** | Bean info class for PredictionAppender. |
| **PredictionAppenderCustomizer** | GUI Customizer for the prediction appender bean |
| **Saver** | Saves data sets using weka.core.converter classes |
| **SaverBeanInfo** | Bean info class for the saver bean |
| **SaverCustomizer** | GUI Customizer for the saver bean |
| **ScatterPlotMatrix** | Bean that encapsulates weka.gui.visualize.MatrixPanel for displaying a scatter plot matrix. |
| **ScatterPlotMatrixBeanInfo** | Bean info class for the scatter plot matrix bean |
| **SerializedModelSaver** | A bean that saves serialized models |
| **SerializedModelSaverBeanInfo** | Bean info class for the serialized model saver bean |
| **SerializedModelSaverCustomizer** | GUI Customizer for the SerializedModelSaver bean |
| **StripChart** | Bean that can display a horizontally scrolling strip chart. |
| **StripChartBeanInfo** | Bean info class for the strip chart bean |
| **StripChartCustomizer** | GUI Customizer for the strip chart bean |
| **TestSetEvent** | Event encapsulating a test set |
| **TestSetMaker** | Bean that accepts data sets and produces test sets |
| **TestSetMakerBeanInfo** | Bean info class for the test set maker bean. |
| **TextEvent** | Event that encapsulates some textual information |
| **TextViewer** | Bean that collects and displays pieces of text |
| **TextViewerBeanInfo** | Bean info class for the text viewer |
| **ThresholdDataEvent** | Event encapsulating classifier performance data based on varying a threshold over the classifier's predicted probabilities |
| **TrainingSetEvent** | Event encapsulating a training set |
| **TrainingSetMaker** | Bean that accepts a data sets and produces a training set |
| **TrainingSetMakerBeanInfo** | Bean info class for the training set maker bean |
| **TrainTestSplitMaker** | Bean that accepts data sets, training sets, test sets and produces both a training and test set by randomly spliting the data |
| **TrainTestSplitMakerBeanInfo** | Bean info class for the train test split maker bean |
| **TrainTestSplitMakerCustomizer** | GUI customizer for the train test split maker bean |
| **VisualizableErrorEvent** | Event encapsulating error information for a learning scheme that can be visualized in the DataVisualizer |

---


|  |  |  |
| --- | --- | --- |
| **Overview** \| **Package** \| Class \| **Tree** \| **Deprecated** \| **Index** \| **Help** \| **Weka's home** | |  |
| **PREV PACKAGE**   **NEXT PACKAGE** | **FRAMES**    **NO FRAMES**     **All Classes** |  |


---
